# Epigenetic pacemaker: closed form algebraic solutions

**DOI:** 10.1186/s12864-020-6606-0

**Published:** 2020-04-16

**Authors:** Sagi Snir

**Affiliations:** Department of Evolutionary & Environmental Biology, Faculty of Natural Sciences, University of Haifa, Haifa, Israel

**Keywords:** Epigenetics, Universal PaceMaker, Conditional Expectation Maximization, Matrix Multiplication, Symbolic Algebra

## Abstract

**Background:**

DNA methylation is widely used as a biomarker in crucial medical applications as well as for human age prediction of very high accuracy. This biomarker is based on the methylation status of several hundred CpG sites. In a recent line of publications we have adapted a versatile concept from evolutionary biology - the Universal Pacemaker (UPM) - to the setting of epigenetic aging and denoted it *the Epigenetic PaceMaker* (EPM). The EPM, as opposed to other epigenetic clocks, is not confined to specific pattern of aging, and the epigenetic age of the individual is inferred independently of other individuals. This allows an explicit modeling of aging trends, in particular non linear relationship between chronological and epigenetic age. In one of these recent works, we have presented an algorithmic improvement based on a two-step conditional expectation maximization (CEM) algorithm to arrive at a critical point on the likelihood surface. The algorithm alternates between a time step and a site step while advancing on the likelihood surface.

**Results:**

Here we introduce non trivial improvements to these steps that are essential for analyzing data sets of realistic magnitude in a manageable time and space. These structural improvements are based on insights from linear algebra and symbolic algebra tools, providing us greater understanding of the degeneracy of the complex problem space. This understanding in turn, leads to the complete elimination of the bottleneck of cumbersome matrix multiplication and inversion, yielding a fast closed form solution in both steps of the CEM.In the experimental results part, we compare the CEM algorithm over several data sets and demonstrate the speedup obtained by the closed form solutions. Our results support the theoretical analysis of this improvement.

**Conclusions:**

These improvements enable us to increase substantially the scale of inputs analyzed by the method, allowing us to apply the new approach to data sets that could not be analyzed before.

## Background

The study of aging and in particular human aging has become a very active field in genomics [1, 2], in particular due to the role of DNA methylation [3]. Methylation serves as an epigenetic marker as it measures the state of cells as they undergo developmental changes [4]. Methylation however continues also beyond the developmental stage, as humans age, notwithstanding at significantly lower rate [5–8]. Therefore DNA methylation serves as a central epigenetic mechanism that helps define and maintain the state of cells during the entire life cycle [9–11]. In order to measure genome-wide levels of DNA methylation, techniques such as bisulfite sequencing and DNA methylation arrays are used [12].

In his seminal paper [13], Steve Horvath defined the term *epigenetic clock*, which later appeared to be a very robust estimation to human age (see e.g. [14]). The scheme is divided into two: children up to age twenty, and adults. A raw estimated age is first calculated by a weighted sum of 353 sites. Then, For the children, this raw age is log transformed to reflect the real chronological age. For adults, this raw age us taken as is. This approach, of using an untransformed epigenetic state as chronological age induces linearity between these two measures. This linearity can be compared to the classical concept from molecular evolutionary known as the as the molecular clock (MC) [15, 16].

**Fig. 1:**
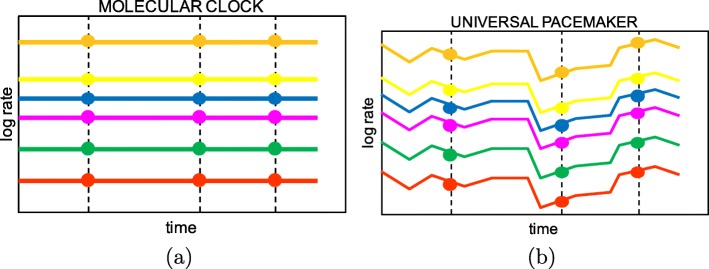
Molecular Clock vs Universal PaceMaker: Solid lines (colors) represent different methylation sites. Vertical (dashed) lines represent time points. Hence dots along dashed lines correspond to (log) methylation rates at that very time point of each methylation site. Under the Molecular Clock (MC) model (left), methylation rates of sites differ among each other but are constant in time. By contrast, under the Universal PaceMaker (UPM) model (right), rates may vary during with time but the pairwise ratio between sites rates remains constant (diference between log rates is constant)

The rate constancy of MC can be relaxed by a mechanism dubbed *the universal pacemaker* (UPM or simply pacemaker - PM) of genome evolution [17–20]. Under the UPM, genes within a genome evolving along a linage, can vary their intrinsic mutation rate in concert with all other genes in the genome. Figure 1 illustrates pictorially differences between the two models - UPM and MC. The UPM mechanism can be adapted from molecular evolution to model the process of methylation.

In a line of works [21–23] we have developed a model borrowing ideas from the UPM to describe methylating site in a body. While the time linearity described above can be perceived as the MC, under the UPM approach, the adapted model assumes that all sites change according to an adjusted time, which is a non-linear function of the chronological time. This paradigm - denoted *the epigenetic pacemaker* (EPM) - becomes appealing for studying age related changes in methylation, where methylating sites correspond to evolving genes.

The first work of the EPM [21] used a simple approach to find the optimal, maximum likelihood, values for the variables sought, what restricted the inputs analyzed to small sizes, and limited the biological inference. In a recent work [22] we have devised a conditional expectation maximization (CEM) algorithm [24] which is an extention to the widespread expectation maximization (EM) algorithm [25]. CEM is applied when optimizing the likelihood function over the entire parameter set simultaneously is hard. The parameters are partitioned into two or more subsets and optimiztion is done separately in an alternating manner. In our specific setting, this partitioning separated the variable set into site variables and *time variables* that are optimized separately in two alternating steps. Here however, we combine the structure of the EPM model with insights from linear algebra, and with the help of symbolic algebra tools (e.g. Sage-math [26]) trace the use of variables through the entire linear algebra stage. The latter allows us to bypass that heavy step completely, resulted in a prominent improvement, both practical and theoretical, and in both running time and memory space. This improvement is complemented by a linear time, closed form solution to the second step, the *time step*, of the CEM. The unification of these two improved steps under a combined high level algorithm as the CEM yields a very fast algorithm that ends in few iterations of the EM algorithm.

The improvements described above give rise to a substantial increase in the scale of inputs analyzed by the method, and enable the applications of the new approach to data sets that could not be analyzed before.

## Methods

### The evolutionary model

Our model includes *mindividuals* and *nmethylation sites* in a genome (or simply sites). We also have for each individual *j* his chronological age *t*_*j*_. The set *t*={*t*_*j*_} is a set of *time periods* of all ages. There is additionally a set of sites *s*_*i*_ that undergo methylation changes, where the *rate* of site *i* is *r*_*i*_. The methylation process starts with an *initial level* at birth $s_{i}^{0}$. Therefore the variables associated with sites, the *site variables*, i.e., *r*_*i*_ and $s_{i}^{0}$ are stored in the vectors of size *n* and the variables associated with individuals, *t*_*j*_ are stored in an *m*-size vector *t*. Henceforth, we will index sites with *i* and individuals with *j*. The variable *s*_*i*,*j*_ measures the methylation level (or status) at site *s*_*i*_ in individual *j* at time *t*_*j*_. Practically, it is the average methylated sites among all that individual’s cells. Under the *molecular clock* model (i.e. when rate is constant over time), we expect: $s_{ij} = s_{i}^{0} + r_{i}t_{j}$. However, we have *noise* component *ε*_*i*,*j*_ that is added and therefore the *observed value*$\hat s_{ij}$ is $\hat s_{ij} = s_{i}^{0} + r_{i}t_{j} + \varepsilon _{i,j}$.

Given the input matrix $\hat S=[\hat s_{i,j}]$, holding the observed methylation level at site *s*_*i*_ of individual *j*, the goal is to find the maximum likelihood (ML) values for the variables *r*_*i*_ and $s_{i}^{0}$ for 1≤*i*≤*n*. Henceforth we define a statistical model under which *ε*_*i*,*j*_ is assumed to be normally distributed, *ε*_*i*,*j*_∼*N*(0,*σ*^2^). In [21], (Lemma 6 thereof) we showed that minimizing the following function, denoted the *residual sum of squares* (or *RSS*), is alent to maximizing the model’s likelihood:

1 $$\begin{array}{@{}rcl@{}}  RSS = \sum\limits_{i\le n} \sum\limits_{j\le m} \left(\hat s_{i,j} - \left(s_{i}^{0} + r_{i}t_{j}\right)\right)^{2}. \end{array} $$

Under such a setting, there is a precise linear algebra solution to this problem, which can be computed efficiently, meaning in time polynomial in the input size. We will elaborate more on this in the sequel.

The more involved model is the EPM model. Under this model, in contrast to the MC, individual’s sites may change their rate at any point in life, and this occurs arbitrarily and independently of their counterparts in other individuals. Nevertheless, when this happens, all sites of that individual change their rate proportionally such that the ratio $\phantom {\dot {i}\!}r_{i}/r_{i^{\prime }}$ is constant between any two sites *i*, *i*^′^ at any individual *j* and at all times. This very property, of strict correlation between site rates at a certain individual, is denoted *the EPM property* and it can be shown [21] that this is equivalent to multiplying the age of individual *j* by the same factor of the rate change. This new age of the individual reflects its biological, or epigenetic, age and hence denoted as the *epigenetic age* (*e-age*), as opposed to the chronological age (*c-age*). Therefore here, we do not just take the given c-age as the individual’s age, rather estimate the e-age of each individual and the c-age is formally ignored (but see implementation comment in real data analysis part). Consequently, in addition to the $s_{i}^{0}, r_{i}$ variates of the MC model, the task under the EPM model is to find the optimal values of $s_{i}^{0}, r_{i}$, and *t*_*j*_ (where, under this model, *t*_*j*_ in the equation represents a weighted average accounting for the rate changes an individual has undergone through life). Below, a solution to this optimization problem is illustrated. The difference between the chronological age and the estimated epigenetic age is denoted as *age acceleration* or *age deceleration* depending on the sign of that difference.

As we here deal primarily with exact slutions to the MC case, the task of comparing between the models - MC and EPM - is beyond the scope of this specific work. However, for the sake of completeness, and since this is the prime goal of the EPM model, we now only mention this. Under the statistical setting we described above, we can use standard tools to compare between the MC and EPM. Recall that under the MC model, a constant rate of methylation at each site is assumed implying time-, or age-, linearity. Conversely, in the alternative, relaxed, model (EPM), there are no such restrictions, and in turn an "epigenetic" age for each individual is estimated. By this definition, the restricted MC solution is contained in the solution set of the relaxed EPM model, and hence cannot exceed the EPM solution. Therefore, in order to compare the approaches, we use the likelihood ratio test (LRT) as explained below.

In order to compare between two competing models, we use a statistical test examining the goodness of fit of the two models. The likelihood ratio test (LRT) assumes one of the models (the null model) is a special case of the other, more general, one. The ratio between the two likelihoods is computed and a log is taken. This quantity is known to distribute as a *χ*^2^ statistic and therefore can be used to calculate a *p*-value. This *p*-value is used to reject the null model in the conventional manner. Specifically, let *Λ*=*L*_0_/*L*_1_ where *L*_0_ and *L*_1_ are the ML values under the restricted and the more general models respectively. Then asymptotically, −2 log(*Λ*) will distribute as *χ*^2^ with degrees of freedom equal the number of parameters that are lost (or fixed) under the restricted model. In our case, it is easy to see that

2 $$  \log \left (\Lambda \right) = -\frac {nm}{2}\log \frac {\widehat{ RSS}_{MC}} {\widehat{ RSS}_{PM}}  $$

where $\widehat { RSS}_{MC} $ and $\widehat { RSS}_{PM}$ are the ML values for RSS under MC and PM respectively. Hence we set our *χ*^2^ statistic as

3 $$  \chi^{2} = {nm}\log \left (\frac {\widehat{ RSS}_{MC}} {\widehat{ RSS}_{PM}} \right).  $$

## Results

In the “Results” section we describe both the technical improvements, that is, the closed form solutions to the CEM step of [22], and subsequently its application to several data sets. We start with a description of the main technical result of this work that is a significant improvement of the previous standard linear algebra solution used in both [21, 22].

### Solving the MC model

#### Overview

As this is the central part of the work, we provide a brief overview of the approach taken. In order to solve the MC model we apply a standard optimization procedure as is shown below. The task is to minimize the RSS (Eq. (1)). An immediate and basic observation, is that for every site *i*, only two variables are involved - $s_{i}^{0}$ and *r*_*i*_, and hence they can be optimized separately from any other site *i*^′^ variables - $s_{i'}^{0}$ and $\phantom {\dot {i}\!}r_{i^{'}}$. Indeed, while the same observation is enough for the *time* variable that we handle inSolving the EPM problem section, as we have here two parameters, the complexity of the polynomial system is significantly larger and we cannot get such a (relatively) simple expression as in Eq. (18). Instead we obtain two polynomials of quadratic degree that should be solved simultaneously. While the latter is manageable for a *numerical* solution, when the time-values are known and the polynomials are rather simple, here the goal is a *closed form* solution, which is substantially more complex as all time-variables exist in the equation. Such a solution forces the use of symbolic algebra tools. Moreover, even using such a tool was not enough to trace the structure of the solution. It is the decomposition to the three steps of the matrix operation, and the definition of special *expanded diagonal matrix*, that allowed us to trace how each such single operation operates on the solution.

#### Proof details

We now describe the proof detail. Denote the maximum likelihood RSS by $\widehat { RSS}$ and we use it for computing *χ*^2^ to obtain confidence values. Every monomial in the RSS stands for an entry in the input matrix $\hat S$, that is $\hat s_{i,j}$, and is of the form:

4 $$ \varepsilon^{2}_{i,j} = \left(\hat s_{i,j} - t_{j} r_{i} -s_{i}^{0}\right)^{2},  $$

where in our case the inputs are the $\hat s_{i,j}$ and *t*_*j*_ and the variables sought are *r*_*i*_ and $s_{i}^{0}$, for every *i*≤*n* (our set of sites).

Critical points in a polynomial are found through partial derivatives with respect to every such variable. These points lie in the 2*n* space where all these partial derivatives vanish simultaneously [27]. The general case problem is NP-hard, and hence no efficient (polynomial time) algorithm exists, let alone a closed form solution. Hence, the polynomial’s roots are normally found via some numerical method.

Here however, the unique structure of the problem permits a more efficient solution. When the residuals are linear in all unknowns, we can use tools from linear algebra to find a solution which have a closed form (given that the columns of the matrix are linearly independent). Under this formalism the optimal (ML) solution is given by the vector $\hat \beta $ as follows:

5 $$  \hat \beta = \left (X^{T}X\right)^{-1} X^{T} y,  $$

where *X* is a matrix over the variable’s coefficients in the problem (also denoted a *design matrix*), *y* is a vector holding the observed values - in our case the entries of $\hat S$, and the RSS equation can be written such that for every row *i* in *X*, $y_{i} - \sum _{j}X_{i,j}\beta _{j}$ is a component in the RSS. Thus, the RSS contains *mn* quadratic terms where *m* and *n* are the number of individuals and sites respectively. Each such term corresponds to an entry in $\hat S$ in the form $\hat s_{i,j} - t_{j} r_{i} -s_{i}^{0}$ where $\hat s_{i,j} $ and *t*_*j*_ are input parameters. This leads to the following observation (stated in [21]):

##### **Observation 1**

**Fig. 2:**
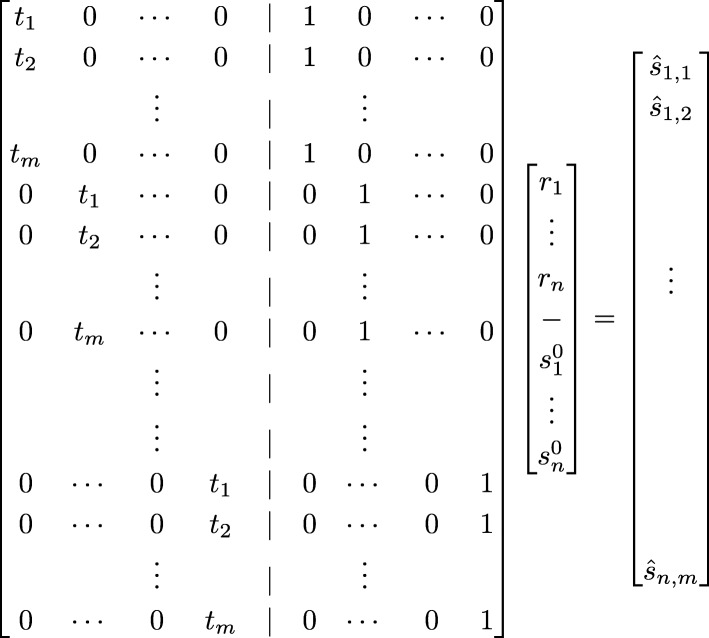
The *mn*×2*n* design matrix *X* that is used in our closed form solution to the MC case. Every row corresponds to a component in the RSS polynomial and the corresponding entries (*i*th and *i*+*n*th) in that row are set to *t*_*j*_ and 1 respectively

([21]) Let *X* be a *mn*×2*n* matrix whose *k*th row corresponds to the (*i*,*j*) entry in *S*, the first *n* variables of *β* are the *r*_*i*_’s and the second *n* variables are the $s_{i}^{0}$’s, and the *im*+*j* entry in *y* contains *s*_*i*,*j*_ (see Fig. *2*). Then, if we set the *k*th row in *X* all to zero except for *t*_*j*_ in the *i*’th entry of the first half and 1 in *i*’th entry of the second half, we obtain the desired system of linear equations (see again illustration for row setting in Fig. *2*).

The likelihood score is calculated by plugging in the values obtained for $ \hat \beta $ in (5) to the likelihood function (or alternatively into the *RSS*).

#### Direct solution of the likelihood function

A standard (algebraic) implementation of Eq. (5) is heavy as it requires the multiplication of the huge 2*n*×*nm* matrix *X* followed by inverting the product matrix, and then another multiplication.Luckily, the specific matrices handled in our case possess substantial structure that is imposed by the EPM framework. Below, by a series of claims we prove the main result of this part, i.e., a fast, closed form solution to Eq. (5) that entirely eliminates the heavy linear algebra machinery.

As the subject is related to matrix multiplication, and also to compare the improvement described here to the previous approach, we provide a brief background to the field. Matrix multiplication and inversion is a classical yet very active subject in computational complexity. Naive multiplication of an *n*×*m* matrix *A* by an *m*×*p* matrix *B* takes *Θ*(*nmp*) time. The Strassen algorithm [28] was the first to go below cubic time. It is based on a recursive subdivision of the matrices in hand and its asymptotic complexity is $O\left (n^{log_{2}7}\right) = O\left (n^{2.807}\right)$. There are several improvements to the Strassen algorithm with the Coppersmith—Winograd algorithm [29] of *O*(*n*^2.375477^) time as the most prominent among them (but see very recent slight improvements [30, 31]). However, even the relatively simple Strassen algorithm requires significantly more space than the naive *Θ*(*nmp*) algorithm, which essentially works with only square matrices (although this is easily solved but with additional complexity), which may turn it to inferior in total. The later improved algorithms incur huge constants that require very large inputs to be competitive, making them practically irrelevant for our case. Therefore, in our comparisons below we compare our algorithm to the naive *Θ*(*nmp*) algorithm.

##### **Theorem 1**

Solving Eq. (*5*) under the EPM framework can be done in *O*(*nm*) time and *O*(*nm*) space in contrast to *O*(*n*^3^*m*) time and *O*(*n*^2^*m*) space under the naive multiplication.

##### *Proof*

Solving (5) incurs four matrix operations. We prove the theorem by analyzing separately each outcome of these steps. The final result is achieved by showing that the vector $\hat \beta $ from (5) can be constructed directly without any of these operations. We start with the matrix product *X*^*T*^*X*.

##### **Lemma 1**

Consider the 2*n*×2*n* matrix *X*^*T*^*X* from Eq. (*5*) where *X* is as defined in Observation *1* and *t*_*j*_ represents the time (age) of individual *j*. Then, the matrix is composed of four *n*×*n* diagonal matrices as follows:

6 $$ X^{T}X = \left(\begin{array}{rr} A & B \\ C & D\\ \end{array}\right),  $$

where (**1**) $A = diag\left (\sum \limits _{i\le m} t_{i}^{2}\right)$, (**2**) $B = C = diag\left (\sum \limits _{i\le m}t_{i}\right)$, and (**3**) *D*=*diag*(*m*). □

We start by showing that each of the four submatrices is diagonal. Consider first the upper left *n*×*n* submatrix *A*. This submatrix is composed of the dot products of columns *k*, ł in *X* for *k*,ł≤*n* in *X*. It is easy to see that the diagonal is non zero since we have non zero columns in *X*. For off-diagonal entries (*k*,ł) in *A*, note that by the construction of *X*, the *k*th column in *X* has non zero entries only in positions (*k*−1)*m*+1 through *km*. Therefore, for any *k*≠ł there is no overlap in the region of non-zero entries and we get zero as the dot product.

For the upper right submatrix, this consists of the dot products of the last (or second) *n* columns of *X* with the first *n* columns. However, note that in terms of zero/non zero entries, these columns are identical (i.e. the *i*th and the *n*+*i*th columns have the same zero/non zero entries for every *i*≤*n*). Therefore the same arguments as before for diagonal/off-diagonal entries hold.

The third, lower left submatrix is identical to the upper right since *X*^*T*^*X* is by definition symmetric.

The last submatrix is obtained by the dot products of the last *n* columns in *X* with themselves. This submatrix is diagonal by the same arguments as the upper left and the fact the last *n* columns are identical to the first *n* columns in terms of zero/non zero entries.

It remains now to prove the value of each entry in these diagonal submatrices. As the first *n* columns have *t*_1_⋯*t*_*m*_ from the (*k*−1)*m*’th to the *km*’th entries for every column *k*, we obtain $\sum \limits _{i \le m} t_{i}^{2}$ at the (*k*,*k*) entry. Similarly, as the last *n* columns have 1 at each entry from the (*k*−1)*m*’th to the *km*’th, for every column *k*, we obtain $\sum \limits _{i\le m} t_{i}$ at the (*k*,*k*) entry in the second and third submatrices, and *m* at the (*k*,*k*) entry in the forth submatrix.

Lemma 1 above showed that the 2*n*×2*n* matrix *X*^*T*^*X* can be constructed directly from the input, without applying matrix multiplication. The next lemma below handles inverting the resulted matrix *X*^*T*^*X*.

##### **Lemma 2**

The 2*n*×2*n* matrix (*X*^*T*^*X*)^−1^ can be factored by the scalar $\Lambda = \frac 1{\left (\sum _{i\le m}t_{i}\right)^{2}-m \sum _{i\le m}t_{i}^{2}} $ and composed of four *n*×*n* blocks:

7 $$ \left(X^{T}X\right)^{-1} = \Lambda \left(\begin{array}{rr} A & B \\ C & D\\ \end{array}\right),  $$

where

1. *A*=*diag*(−*m*),
2. $B = C = diag\left (\sum \limits _{i\le m}t_{i}\right)$,
3. and $D = diag\left (-\sum \limits _{i\le m}t_{i}^{2}\right)$.

We note though that the constant *Λ* is part of the diagonals of the original matrix (*X*^*T*^*X*)^−1^.

##### *Proof*

We use the Woodbury matrix identity ([27], p.93) stating that a partition of a matrix into four disjoint submatrices satisfies:

8 $$ {\begin{aligned} {\left (A' -B'D'^{-1}C'\right)^{-1}= A'^{-1}+A'^{-1}B'\left (D'-C'A'^{-1}B'\right)^{-1}C'A'^{-1}}, \end{aligned}}  $$

provided *A*^′^ and *D*^′^−*C*^′^*A*^′^^−1^*B*^′^ are invertible. It is important to note that *A*^′^,*B*^′^,*C*^′^ and *D*^′^ are general and have no relationship to the specific matrices we analyze here, specifically to *A*, *B*, *C* and *D*. By the Woodbury matrix identity and the block-wise inversion formula [32] we have

9 $$ {\begin{aligned} \left(\begin{array}{cc} A & B \\ C & D\\ \end{array}\right)^{-1}= \left(\begin{array}{cc} \left (A -BD^{-1}C\right)^{-1} & -\left (A -BD^{-1}C\right)^{-1}BD^{-1} \\ -D^{-1}C\left (A -BD^{-1}C\right)^{-1} & D^{-1}+D^{-1}C\left (A -BD^{-1}C\right)^{-1} BD^{-1}\\ \end{array}\right) \end{aligned}}  $$

Throughout the proof, we work with two matrices, the “input matrix” *X*^*T*^*X* that we invert, and the inverted matrix (*X*^*T*^*X*)^−1^ that is the “output”. As we work block-wise, we use the letters *A* to *D* to denote the blocks of the result, output matrix, and the letters *E* to *H* for the input matrix. Accordingly we have

10 $$  X^{T}X= \left(\begin{array}{rr} E & F \\ G & H\\ \end{array}\right),  $$

and by the Woodbury identity we first need to show our matrices *E* and *H*−*GE*^−1^*F* are invertible. By Lemma 1, $E = diag \left (\sum \limits _{i\le m}t_{i}^{2}\right)$ and hence invertible with $E^{-1} = diag \left (\frac 1{\sum \limits _{i\le m}t_{i}^{2}}\right)$. Similarly, we also have *H*, *G*, and *F* diagonal matrices, so *H*−*GE*^−1^*F* is diagonal and hence invertible.

Next we note that

11 $$  \left (E -FH^{-1}G\right) = diag\left (\sum\limits_{i\le m}t_{i}^{2} - \frac {\left (\sum\limits_{i\le m}t_{i}\right)^{2}}{m} \right)  $$

and therefore its inverse is

12 $$\begin{array}{@{}rcl@{}}  \left (E -FH^{-1}G\right)^{-1} &=& diag\left (\frac m{m\sum\limits_{i\le m}t_{i}^{2} - {\left (\sum\limits_{i\le m}t_{i}\right)^{2}}} \right)  \\  \\ & = & \Lambda \cdot diag (-m). \end{array} $$

Now, it can be seen that *Λ* appears in every submatrix of the inverted matrix (*X*^*T*^*X*)^−1^ but each time with different multipliers. As all our matrices are diagonal, they commute and also their sum and products are diagonal, so we only need to take care of the scalars in the diagonals.

For *A* we have:

13 $$\begin{array}{@{}rcl@{}}  A &=& \left (E -FH^{-1}G\right)^{-1} (by Eq.~(9))  \\  \\ & = & \Lambda \cdot diag (-m). \makebox[2in]{(by Eq.~(12)) } \end{array} $$

For *B* we have

14 $$\begin{array}{@{}rcl@{}}  B &=& - \left (E -FH^{-1}G\right)^{-1} FH^{-1} {(by Eq.~(9))}  \\  \\ &=& -AFH^{-1}. \end{array} $$

Now, by Lemma 1 we have $F=diag \left (\sum \limits _{i\le m}t_{i}\right)$ and *H*=*diag*(*m*), therefore from (14) and (13) above we get

15 $$\begin{array}{@{}rcl@{}}  B &=& \Lambda \cdot diag \left (\sum\limits_{i\le m}t_{i}\right). \end{array} $$

For *C* we have

16 $$\begin{array}{@{}rcl@{}}  C &=& -H^{-1}G\left (E -FH^{-1}G\right)^{-1} \qquad \qquad \qquad \text{(by Eq.~(9))}  \\ &=& -H^{-1}GA  \\ &=& -AGH^{-1} \qquad\qquad \text{(as diagonal matrices commute)} \\ &=& -AFH^{-1} \qquad\qquad\qquad(\text{as } F=G \text{ by Lemma~1})  \\ &=& B \qquad \qquad \qquad \quad\text{(by Eq.~(14))}  \\ &=& \Lambda \cdot diag \left (\sum\limits_{i\le m}t_{i}\right). \end{array} $$

It remains to prove for *D*. By Eq. (9) we have

17 $$\begin{array}{@{}rcl@{}} D &=& H^{-1}+H^{-1}G\left (E -FH^{-1}G\right)^{-1} FH^{-1}  \\ D &=& H^{-1}+H^{-1}GAFH^{-1} \qquad \qquad \qquad\text{(again by Eq.~(9))}  \\ &=& \frac 1m +\frac 1m \sum\limits_{i\le m}t_{i} A\sum\limits_{i\le m}t_{i}\frac 1m \qquad \qquad \quad \text{(by Lemma~1)}  \\ &=& \frac 1m +\frac 1m \left (\sum\limits_{i\le m}t_{i}\right)^{2} m\Lambda\frac 1m  \\ &=& \frac 1m \left (1+\frac {\left (\sum\limits_{i\le m}t_{i}\right)^{2}}{m\sum\limits_{i\le m}t_{i}^{2} - \left (\sum\limits_{i\le m}t_{i}\right)^{2}} \right)  \\ &=&\left (\frac { \sum\limits_{i\le m}t_{i}^{2}}{m\sum\limits_{i\le m}t_{i}^{2} - \left (\sum\limits_{i\le m}t_{i}\right)^{2}} \right)  \\ &=& \Lambda \cdot diag \left (\sum\limits_{i\le m}t_{i}^{2} \right) \end{array} $$

□

Now that we ended with the structure of the matrix (*X*^*T*^*X*)^−1^, we can move to the third and last step of our derivation of the matrix (*X*^*T*^*X*)^−1^*X*^*T*^. This matrix is not square anymore and cannot be decomposed into square diagonal matrices as before. Instead, it can be described as an (*n*,*m*)*-expanded diagonal* matrix which is originated from a *n*×*n* diagonal matrix whose each entry was duplicated *m* times to the right. Therefore the number of rows is *nm* instead of *n*, and the “diagonal” entries are a band spanning *m* entries. We care only for the 0-entries and allow the *m* “diagonal” entries to attain any value. Formally,

##### **Definition 1**

An (*n*,*m*)*-expanded diagonal matrix* is an *n*×*mn* matrix in which for every row *i*, all entries before the *im* entry, and after the (*i*+1)*m*−1 entry, must attain zeros.

##### **Lemma 3**

The 2*n*×*mn* matrix (*X*^*T*^*X*)^−1^*X*^*T*^ is composed of upper and lower (*n*,*m*)-expanded diagonal matrices, *U*, *L* as follows:(**1**) $[U]_{k,l} = -mt_{l-(k-1)m}+\sum \limits _{i\le m} t_{i}$, for (*k*−1)*m*≤*l*≤*km* and 0 otherwise, (**2**) $[L]_{k,l} = t_{l-(k-1)m}\sum \limits _{i\le m} t_{i} - \sum \limits _{i\le m} t_{i}^{2} $ for (*k*−1)*m*≤*l*≤*km* and 0 otherwise.

##### *Proof*

First, we note that by the definition of *X* (and therefore of *X*^*T*^), every *m* consecutive columns in *X*^*T*^ are of the form $\phantom {\dot {i}\!}(0,\ldots,0,t_{i^{'}},0,\ldots,0,1,0,\ldots,0)$ where the location $\phantom {\dot {i}\!}t_{i^{'}}$ in the vector is the number of the *m*-tuple (i.e. first *m* columns, second, etc). The identity of $\phantom {\dot {i}\!}t_{i^{'}}$ (i.e. *i*^′^) is the location of it in the tuple (that is *t*_1_ is the first and *t*_*m*_ last in the tuple). It should be noted that the index *i*^′^ here is entirely unrelated to *i* in the sums along the proofs and represents an independent index. Now, the location of the ’1’ in the vector, is the same only that counting starts from the middle of the vector (refer again to Fig. 2). Furthermore, by Lemma 2, (*X*^*T*^*X*)^−1^ is composed of four *n*×*n* block diagonal matrices. We therefore analyze separately the upper *n* rows that correspond to the upper *n* coordinates of each column in the result (*X*^*T*^*X*)^−1^*X*^*T*^, that is, the first part of the claim. The upper *k* row, by Lemma 2, is of the form $(0,\ldots,0,-m,0,\ldots,0,\sum t_{i},0,\ldots,0)$ where the non zero values appear in the diagonals, i.e. *k* and *n*+*k* (recall the length of the row is 2*n*). Since this *k*th row is non zero only at these two diagonals the non zero entries in *k*th row in the product (*X*^*T*^*X*)^−1^*X*^*T*^ are from the columns in *X*^*T*^ where *k*th entry is non zero. This holds exactly and only for the *k*th *m*-tuple of columns. Since the form of the *j*th column in the *k*th *m*-tuple of rows is (0,…,0,*t*_*j*_,0,…,0,1,0,…,0) the product of $-mt_{j}+\sum \limits _{i\le m}t_{i}$ is obtained as required. This completes the first part of the claim.

The result for the lower *n* rows in (*X*^*T*^*X*)^−1^*X*^*T*^ (second part of the claim) is obtained similarly. Note that here, by Lemma 2, the two diagonals have $\sum \limits _{i \le m}t_{i}$ and $\sum \limits _{i \le m}t_{i}^{2}$, hence the *k*th row in the lower half of (*X*^*T*^*X*)^−1^ has $\sum \limits _{i \le m}t_{i}$ and $-\sum \limits _{i \le m}t_{i}^{2}$ at positions *k* and *n*+*k*. Therefore, at the product (*X*^*T*^*X*)^−1^*X*^*T*^ we will have non zero values only for columns ł for (*k*−1)*m*≤ł≤*km* and the inner product will be $t_{j}\sum \limits _{i \le m}t_{i}-\sum \limits _{i \le m}t_{i}^{2}$ as required. □

After settling the structure of the (*X*^*T*^*X*)^−1^*X*^*T*^ matrix, our goal is to produce the results vector *β* that contains the values of our missing variables - the rate vector *r* and the starting states *s*^0^. Recall that the matrix (*X*^*T*^*X*)^−1^*X*^*T*^ is of size 2*n*×*mn* and therefore any naive multiplication of a vector with it will incur time of *Ω*(*n*^2^*m*) which will turn all our previous efforts futile. However, as that matrix is sparse, and importantly, we know the values of the entries and their location without deriving the actual matrix, we can do much better. The following observation formalizes precisely the arguments above.

##### **Observation 2**

The multiplication (*X*^*T*^*X*)^−1^*X*^*T*^*y* can be done with only 2*nm* scalar multiplications.

##### *Proof*

We first note that the matrix (*X*^*T*^*X*)^−1^*X*^*T*^ has only *m* non-zero entries in each row and moreover, their exact location is known and their value is dependent only on that location. That suggests that we do not need to hold the matrix or even some advanced data structure to keep sparse matrices. Instead, in each row *k* of (*X*^*T*^*X*)^−1^*X*^*T*^ we find the entries in *y* that are affected, we calculate the value in the matrix (this is determined solely by the indices of that entry) and perform the multiplication with the corresponding value in *y*. As the value at the entry is a multiplication of the appropriate time value *t*_*k*_ and the values $\sum \limits _{i \le m}t_{i}$ and $\sum \limits _{i \le m}t_{i}^{2}$, we can compute the latter two once in advance and use them throughout the matrix multiplication.Therefore we have 2*m* multiplication at the preprocessing stage to calculate $\sum \limits _{i \le m}t_{i}$ and $\sum \limits _{i \le m}t_{i}^{2}$ and 2*nm* multiplication for the actual matrix multiplication. □

By Lemma 3 we can calculate in advance all entries of that matrix and then multiply. Therefore we can conclude,

##### **Corollary 1**

The result vector *β* can be computed directly without all the heavy linear algebra machinery.

This concludes the proof of the main Theorem 1.

### Solving the EPM problem

Theorem 1 gives a closed form, non linear algebraic, solution to the MC problem. However, under the EPM model, we cannot apply the same tools as in the MC model as the set of times *t*_*j*_’s also need to be estimated, forming a non linear function in the RSS polynomial. Hence we ought to seek a heuristic solution that will provide a sound result in reasonable time and for non trivial data, as the formulation from the step above does not hold. The Conditional Expectation Maximization (CEM) [24] algorithm that we devised in [22] addresses this challenge by subdividing the maximization step into two steps in which at each step the likelihood function is maximized over a subset of the variates conditional on the values of the rest of the variates.

As our set of variates under the EPM formulation is augmented with the times (individual’s epigenetic ages) it is now composed of the set of sites, starting states, site rates, and times. Hence, in order to arrive at a local optimum point, we partition the set of variates into two: one is the set of rates and start states, and the other is the set of times. The CEM algorithm optimizes separately each such set by alternating between two steps: the *site step* in which the site specific parameters, rate and starting state, are optimized, and *time step* in which individual’s times are optimized. At every such step an increase in the likelihood is guaranteed, until a local optimum is reached.

In our specific case, it remains to show how we optimize the likelihood function at each step. Note, that one of the sets of variates is exactly the set we solved for under the MC formulation - the set of rates and site start states. For this set, we already have a very fast algorithm that is provably correct by Theorem 1. We now show how maximization is done for the other set of variates - the set of times *t*_*j*_.

#### **Lemma 4**

The maximum likelihood value for the time *t*_*j*_ is given by the following closed form rational function:

18 $$  t_{j} = \frac{\sum\limits_{i\le n} r_{i}\left(\hat s_{i,j} - s_{i}^{0}\right)}{\sum\limits_{i\le n} r_{i}^{2}}.  $$

#### *Proof*

Recall that the likelihood function (i.e. the *RSS*) is a polynomial over the set of variates,

19 $$\begin{array}{@{}rcl@{}} RSS = \sum\limits_{i\le n} \sum\limits_{j\le m} \left(\hat s_{i,j} - \left(s_{i}^{0} + r_{i}t_{j}\right)\right)^{2}. \end{array} $$

In the current case, by the CEM algorithm, we freeze all the variates save for *t*_*j*_’s, and therefore can treat them as constants and optimize only for the *t*_*j*_’s. This is done by finding the partial derivatives of the likelihood function, with respect to the variates to be optimized, and then solving these equations jointly (i.e. finding the set of values under which all these partial derivatives vanish.The above sentences are generic and apply to any polynomial. However, our formulation posses a special structure that provides for the closed form of (18). Specifically, note that every term in the *RSS* contains exactly a single time variate *t*_*j*_. This implies that after derivation, we will have a polynomial only with that *t*_*j*_ and since the *RSS* is quadratic in *t*_*j*_, the derivative will be linear in *t*_*j*_. Denote *S*_*j*_ the sum of the terms in the *RSS* associated with *t*_*j*_. Then $S_{j} = \sum \limits _{i\le n} \left (\hat s_{i,j} - \left (s_{i}^{0} + r_{i}t_{j}\right)\right)^{2},$ and after derivation in *t*_*j*_ we get $S_{j}' =\sum \limits _{i\le n} 2\left (r_{i} s_{i}^{0}-r_{i}\hat s_{i,j} + r_{i}^{2}t_{j}\right)$.

After equating to zero and solving for *t*_*j*_ Eq. (18) follows. □

#### **Corollary 2**

The time optimization step can be done in time *O*(*nm*).

#### *Proof*

By Eq. (18) we see that for each *t*_*j*_ we have *n* summations and each summation consists of a single multiplication. As this applies to any *t*_*j*_, the result follows. We note that the quantity $\sum r_{i}^{2}$ can be computed independently as a preprocessing step. □

For the sake of completeness, we here describe the full high-level CEM algorithm from [22]. The algorithm alternates between the two steps, the *time step* and the *site step* as long as an improvement greater than a threshold *δ*_*CEM*_ is attained. We use *RSS*(*p*) to denote the evaluation of the polynomial *RSS* with a set of parameters *p*.


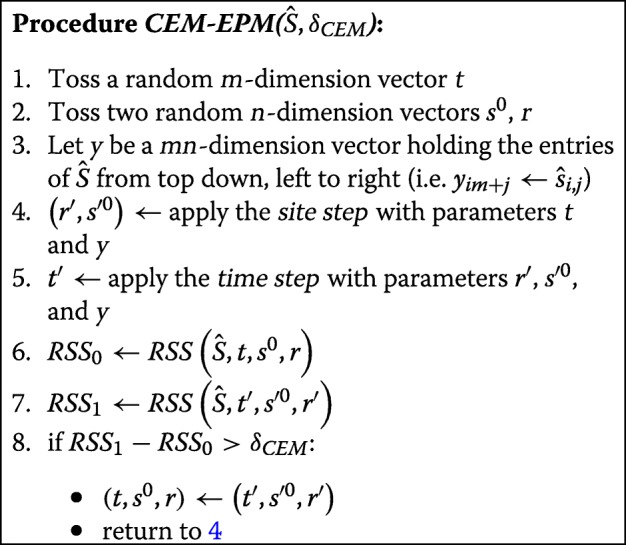


### Real data results

We incorporated the improved procedure into the conditional expectation maximization (CEM) procedure outlined in [22] and implemented it in code. In order to demonstrate the speedup of the improvement, we applied the code under two modes to real methylation data from six data sets. The first mode runs the CEM algorithm but when the MC stage is done via the four linear algebra matrix operations as incurred by Eq. (5) under the standard Python math library implementation - *Numpy*. We note though that Python has a special function for least squares in its linear algebra package of Numpy- linalg.lstsq - but at this stage, we chose to use the manual algebraic operations, and deferred this use to later. In the second mode we simply used the closed form solutions as described in the proof of Theorem 1. The *time step* was performed identically in both modes, as depicted by Eq. (18). For fast convergence of the iterative CEM algorithm, we used the input chronological age as a starting guess for the hill climbing. All data sets were processed by a Macbook laptop with a 2.7 GHz Intel Core i5 processor with 8GB memory.

The data sets differ mainly by their sizes - number of individuals. As in previous studies, for all data sets, we chose the 1000 sites providing the largest Pearson correlation with time [21, 22]. Our first data set is the GSE87571, from human blood taken from 366 individuals [33]. The second data set is from GSE40279, consisting of 656 blood samples from adults [34]. The Next data set is the GSE64495, also from blood samples of 113 individuals [35]. Then, the GSE60132, taken from peripheral blood samples of 192 individuals of Northern European ancestry [36]. The fifth data set is the GSE74193, consisting of 675 samples from brain tissues from before birth to old age [37]. Our last data set is the GSE36064 data set of blood samples taken from 78 children of ages ranging from one year to 16 [38].

The analysis of the results obtained is highly involved and concerns with the ages of individuals in the data sets, therefore requires further analysis of the properties exhibited by the epigenetic age. As this work focuses more on the technical aspects of the algorithm and not on the epigenetic aspects involved, the latter is beyond the scope of the current work. Hence it is deferred to a later publication, and we here focus only on running times.

The results of our runs are depicted in Table 1. We see that for the two largest data sets, GSE40279 and GSE74193, with 656 and 675 individuals respectively, the naive linear algebra implementation could not terminate and we associate this also to the space consumption of this step and less to the time complexity (or to their combination). For the four other data sets, we see a speed up of above 300 with exception for GSE60132 with speedup of 192. These results stand in agreement with our prediction of a linear speedup as suggested by our theoretical results.

**Table 1:** Detailed experimental results. The columns, from left to right are: data set id, description (tissue, ages), # individuals, running time (minutes) under the closed form - T(CF), running time (minutes) under the linear algebra operations - T(LA), residuals sum of square (RSS) under MC, RSS under EPM, *χ*^2^, Degree of freedom for *χ*^2^. All *p*-values of *χ*^2^ are below 10^−6^

Data Set	Description	n	T(CF)	T(LA)	RSS_MC_	RSS_EPM_	*χ*^2^	DF
GSE87571	Adults, Blood	366	2.4	745	29.9145	25.6283	2716.8	366
GSE40279	Adults, Blood	656	5.63	NA	142.265	115.3552	13479.5	656
GSE64495	Human, All Ages, Blood	113	0.7	254	258.333	203.851	26765.0	113
GSE60132	Human, All Ages, Blood	192	1.8	346	19498.871	17122.519	24952.7	192
GSE74193	Human, All Ages, Brain development	675	7.16	NA	1614.285	712.837	551740.8	675
GSE36064	Children Blood	78	0.52	193	166.774	148.41	9099.3	78

Our next experiment was to check the effect of number of sites - *n*. Here, we chose to use the improved least square package of Numpy. We chose only a single data set from the above for this experiment, the GSE40279, of 656 blood samples from adults [34]. The number of sites selected were 50, 100, 500, 1000, and 5000. The running times obtained were 10 seconds, 1.23 minutes, 47 minutes, and 300 minutes, for the 50, 100, 500, 1000, sites respectively. For the 5000 sites the Numpy least squares could not terminate while our improved version of closed form solution ended in less then 10 minutes.

## Conclusions and discussion

In this work we showed a closed form rational function solution to the epigenetic pacemaker problem. This solution replaces the cumbersome linear algebra step employed in the procedure for solving the likelihood function under the molecular clock (MC) model. Under the EPM model, such a solution can be used as a subroutine in the conditional expectation maximization approach we have developed in our previous work. Under this approach the MC problem is solved in a *site step*, that is applied interchangeably to a *time step*, until a local optimum point is reached. Both steps, as we showed here are done accurately via closed form solutions.

We demonstrated the speedup induced by this improvement by applying it to six data sets of considerable sizes. The analysis used the CEM algorithm described above but with and without the closed form algebraic solutions. We showed that for data sets of moderate sizes, a speedup of about 300 fold is achieved. Notwithstanding, for the larger data sets of more than 600 individuals, the linear algebraic solution could not run, and we associate this also to the space improvement of the closed form solution.

Finally and importantly, the use of advanced tools such as symbolic algebra has value beyond the mere algorithmic improvements illustrated here, rather it grants a deeper understanding of the internals of the model that cannot be achieved otherwise.As a future research direction, we seek to further understand the likelihood surface. This understanding will not only teach us about the degeneracy of this surface with regard to multiple ML points, but also the relationship between them and what invariants they satisfy. In the biological realm, an immediate goal is to provide a rigorous analysis of the trends we see in aging - is there a trend in the population towards non linear (i.e. constant) ratio between epigenetic age versus chronological age.

## Abbreviations

The following abbreviations are used in this manuscript. CEM: conditional expectation maximization
EPM: epigenetic pacemaker
LRT: likelihood ratio test
MC: molecular clock
ML: maximum likelihood
RSS: residual sum of squares
